# Recent Dental Research
*Paper read at a meeting of the Bath and Bristol Branch of the British Medical Association, 30th April 1930.


**Published:** 1930

**Authors:** R. H. McKeag


### RECENT DENTAL RESEARCH.*

BY

R. H. McKeag, M.A., M.B., B.Ch., B.A.O.,
B.Dent.Sc.

---

Dental research is greatly handicapped by the fact that the training of dental students has necessarily to contain so vast an amount of the purely mechanical. Secondly, the almost complete absence of full-time teaching or other dental appointments at the universities is a bar to research. Thirdly, the lack of co-operation between the medical specialists and the dentists is a great handicap to progress. In hospital the physician can on any case obtain the services of a whole team of specialists, whereas the dental surgeon is usually lucky if he can get the assistance of the radiographer alone.

Along the commercial side, research, both by the big manufacturing firms and by individual dentists, has been extensive.

In orthodontia specialists have arisen, and consequently extensive investigations are at last being made as to the causes of mouth deformities and displacement of teeth, but comparatively little definite information has so far been discovered.

To the work done on dental sepsis as a factor in systemic disease I will refer but briefly, as it is only a few months since Mr. Fawn addressed this branch in Bristol largely on that subject. Dr. Bulleid, working

* Paper read at a meeting of the Bath and Bristol Branch of the British Medical Association, 30th April 1930.

under the Medical Research Council, has made cultures from a very large number of cases of extracted teeth with rarefied areas, chronic abscesses and granulomata. He found streptococci of all types, but chiefly the non-hæmolytic or viridans type, staphylococci, chiefly albus but also aureus, and also a diphtheroid bacillus—Bacillus xerosis. The ærobic and anærobic findings were identical, except that in some cases of granulomata he was able to isolate the Bacillus fusiformis and Leptothrix buccalis anærobically. From the finding of the latter he deduces that repair by calcification may be possible in the granulomata. In no case did he find the proteolytic organisms which are responsible for the bad smell in septic root canals. From his cultures he inoculated mice, and found that the most virulent organisms were those from areas of rarefaction. The cultures from granulomata (which incidentally are the cases which show up best in X-ray photographs) were comparatively innocuous. He further compared his findings with the X-ray photographs taken before extraction, and concludes thus : "No dead tooth can be permanently sterilized whatever technique is adopted, and the teeth least likely to show gross apical sepsis are those in which the root canals are left unfilled or only partially filled."

The subject of residual sepsis is much discussed in dental circles at present, but I can find little of value as yet published about it. The idea is that the mere extraction of a septic tooth, particularly in the lower jaw, may not be sufficient to remove the infection caused by the tooth. There may, of course, be a root remaining, but apart from that there may also be left an area of infected bone, which may continue to feed infection into the bloodstream.

Coming next to the cause of dental caries and its prevention, we find that there are three schools of thought. The first discovered many years ago that carbohydrate decomposition produced an acid which dissolved the enamel, and thereby enabled the mouth bacteria to attack the dentine. They therefore started the slogan, " Clean teeth do not decay," and, content with that, went no further, except to attack investigators along other lines with a surprising virulence. Unfortunately many people who have taken every possible precaution to keep their teeth clean suffer severely from caries. Also one sometimes meets complete freedom from decay in the mouth of a person who has taken no precaution to clean his teeth.

Secondly there is the theory advanced mainly by Broderick of Bournemouth, that in any tendency towards body acidosis, calcium is taken from the tissues and saliva to neutralize the excess acid, and this produces a degree of acidity in the mouth which renders possible an electrical reaction, causing loss of calcium from the enamel of the teeth. In practice what would happen is that there would be a highly acid focus due to food decomposition, let us say in a depression between the cusps of a molar. There would also be an acid of lesser strength (the saliva) bathing both the tooth and this focus. The strong acid frees ions of calcium from the tooth, which are of the same sign as the tooth and are therefore repelled from it. The result is loss of calcium enamel. In support of this theory is (1) the undoubted fact that an acid saliva is usually to be found where there is rapid caries ; (2) various forms of indigestion are followed by decay ; (3) fevers and other diseases in children which produce an acid saliva usually also produce caries. So far, however, no conclusive statistics have been produced. In 1927 and in 1928–29 I carried out a series of tests on the acidity or hydrogen-ion concentration of the salivas of a large number of boys, the results of which were given to dental meetings at Cheltenham and Exeter, and afterwards published in the *British Dental Journal.* While these tests did not entirely support Mr. Broderick's views, they did not disprove them. Where, I think, Broderick makes a mistake is in assuming that an acid saliva means one with less calcium than a more alkaline saliva. Lee Pattison raised the calcium content of a group of patients by giving them a diet rich in milk, cod liver oil and egg, but reports that the pH bore no relation to the amount of salivary calcium.

The vitamin school, as it is called, relies on actual clinical findings rather than on a definite theory. The chief worker here is Mrs. Mellanby, who has spent eleven years experimenting on the teeth of animals by means of diet. She has also made experiments on children, but the only one so far published was to some extent spoiled by the introduction of too many differing factors in the diets. She has undoubtedly proved that developing teeth are adversely affected and rendered prone to future decay by lack of vitamin D in the diet of the pregnant mother and the very young offspring. The moot point is as to whether teeth when once erupted can be influenced by diet in other ways than mechanically. Until very recently it was taught in every dental school that enamel, once formed, could only be affected by mechanical stresses or chemical action of acids; that under no condition could any improvement in its condition be effected. Now the experiments of Fish and Caush lead, shall I say, the less sceptical of us to believe that there are evidences of a lymph supply in the enamel, and, to my mind, clinical observation points to a likelihood of the enamel acting in a similar way to most parts of the body in resisting disease.

To test the action of vitamin D and also of parathyroid extract on the calcification of erupted teeth, or at least their action in preventing decay, I divided all the boys at the Somerset Boys' Home who were expected to remain there for a year into three groups as equal in every way as I could. Let us call these groups A, B and C. A was the control, B were given two "ostelin" pills a day for twelve months; C were given the same number of "ostelin and parathyroid" pills. The "ostelin" tablets contained ostelin 4 m., calcium glycerophosphate 2 gr.; the "ostelin and parathyroid" tablets the same plus 1/40 gr. of desiccated parathyroid extract. The tablets were supplied free of cost at my request by Messrs. Nathan, the manufacturers, but the experiment was entirely in my hands, and had no other connection with the firm. For certain reasons the disbandment of the school began before the end of the year of test, and therefore my groups which started twenty-four strong were reduced in numbers. All mouths were carefully charted before and after the year of experiment, and to ensure absolute avoidance of bias I obtained the assistance of Mr. C. M. John, L.D.S., in the charting. The results you see on this chart:—

| | Number of boys in group. | Number showing fresh decay. | Percentage. showing fresh decay. | Number of teeth showing fresh decay. | Average number of teeth decayed per boy. |
|---|---|---|---|---|---|
| Group A .. .. (Control) | 20 | 10 | 50% | 25 | 1·25 |
| Group B .. .. (Ostelin) | 16 | 7 | 44% | 13 | ·81 |
| Group C .. .. (Ostelin and parathyroid) | 19 | 6 | 32% | 8 | ·42 |

Leaving out decimals, it shows that for every three teeth attacked by caries among those on the ordinary diet, only two suffered when ostelin was given, and only one when parathyroid was added to the ostelin. I maintain from these figures that, after making due allowance for errors in averaging somewhat small numbers, it would appear that the extra supply of vitamin D was undoubtedly of value in reducing the incidence of dental decay. I make this assertion so definitely, partly because it is supported by other clinical observation, and also because Mrs. Mellanby's work corroborates it. Sufficient testing of parathyroid extract has not as yet been made to warrant my being dogmatic on its value.

In conclusion may I appeal for a closer co-operation between the medical and dental professions? It should be possible for the dental surgeon on finding rapid caries in the mouth of a patient to send that patient to his or her doctor, in order that the general condition of which I believe the caries to be only a symptom might be put right. It is not the function of the dentist to act as a medical adviser. Also the dentist in his effort to solve the problems of dental disease should have the wholehearted assistance of the bacteriologist, pathologist and radiographer, and also the general practitioner.

To sum up the points I have tried to make in so short a time:

1. No mouth can be considered free from a focus of infection if it contains a dead tooth, a root or unerupted tooth, or an area of bone containing residual sepsis. To ascertain whether any of these is present an X-ray photograph is necessary, but the most easily observed lesions are by no means necessarily the most dangerous.

2. The pregnant mother should be amply supplied with calcium and vitamin D, *i.e.* milk and cod-liver oil.

3. These two articles of diet are even more necessary to the developing child, and may be of value to the adult.

4. The correction of an acidosis or even a tendency towards an acidosis may be of considerable value in preventing dental decay.

